# Adiponectin Exerts Peripheral Inhibitory Effects on the Mouse Gastric Smooth Muscle through the AMPK Pathway

**DOI:** 10.3390/ijms21249617

**Published:** 2020-12-17

**Authors:** Eglantina Idrizaj, Rachele Garella, Silvia Nistri, Alfonso Dell’Accio, Emanuele Cassioli, Eleonora Rossi, Giovanni Castellini, Valdo Ricca, Roberta Squecco, Maria Caterina Baccari

**Affiliations:** 1 Department of Experimental & Clinical Medicine, Section of Physiological Sciences, University of Florence, 50134 Firenze, Italy; rachele.garella@unifi.it (R.G.); mcaterina.baccari@unifi.it (M.C.B.); 2 Department of Experimental & Clinical Medicine, Research Unit of Histology & Embryology, University of Florence, 50139 Firenze, Italy; silvia.nistri@unifi.it; 3 Department of Biomedical, Experimental & Clinical Sciences “Mario Serio”, University of Florence, 50134 Firenze, Italy; alfonso.dellaccio@unifi.it; 4 Department of Health Sciences, Psychiatry Unit, University of Florence, 50134 Firenze, Italy; emanuele.cassioli@unifi.it (E.C.); e.rossi@unifi.it (E.R.); giovanni.castellini@unifi.it (G.C.); valdo.ricca@unifi.it (V.R.)

**Keywords:** adiponectin, cell signaling, AMPK, AdipoR1, gastric fundus, mechanical activity, cell excitability

## Abstract

Some adipokines, such as adiponectin (ADPN), other than being implicated in the central regulation of feeding behavior, may influence gastric motor responses, which are a source of peripheral signals that also influence food intake. The present study aims to elucidate the signaling pathways through which ADPN exerts its actions in the mouse gastric fundus. To this purpose, we used a multidisciplinary approach. The mechanical results showed that ADPN caused a decay of the strip basal tension, which was abolished by the nitric oxide (NO) synthesis inhibitor, L-N^G^-nitro arginine (L-NNA). The electrophysiological experiments confirmed that all ADPN effects were abolished by L-NNA, except for the reduction of Ca^2+^ current, which was instead prevented by the inhibitor of AMP-activated protein kinase (AMPK), dorsomorphin. The activation of the AMPK signaling by ADPN was confirmed by immunofluorescence analysis, which also revealed the ADPN R1 receptor (AdipoR1) expression in glial cells of the myenteric plexus. In conclusion, our results indicate that ADPN exerts an inhibitory action on the gastric smooth muscle by acting on AdipoR1 and involving the AMPK signaling pathway at the peripheral level. These findings provide novel bases for considering AMPK as a possible pharmacologic target for the potential treatment of obesity and eating disorders.

## 1. Introduction

Adipokines are molecules secreted by the white adipose tissue and are involved in a wide range of functions through endocrine, paracrine, and autocrine mechanisms [1,2]. Among the different functions, some adipokines are implicated in the central regulation of feeding behavior and also affect gastrointestinal motor phenomena [3], which are known to be peripheral signals that are able to influence food intake through the gut–brain axis [4].

In this regard, we recently observed that adiponectin (ADPN), an adipocytes-released peptide that plays a role in the regulation of food intake at the central level, was also able to exert a neuromodulatory inhibitory effect in strips from the mouse gastric fundus [5]. In agreement with the mechanical studies [5], our preliminary electrophysiological investigations indicated that the hormone also depressed gastric smooth muscle cell (SMC) excitability [6]. Different ion channels are present in the gastric smooth muscle playing key roles in the modulation of SMC excitability and contraction. Particularly, L-type calcium channels have been reported in both fundal [6,7] and antral gastric strips isolated from rats [8] as well as in antral myocytes of guinea pigs [9]. Notably, voltage-dependent L-type Ca^2+^ channels are the backbone of excitation–contraction coupling in the gut [10,11]. However, gastrointestinal SMCs express additional T-type calcium channels that may contribute as a factor in delivery of Ca^2+^ to the contractile apparatus in some cells [12]. Many K^+^ channels are also expressed in gastrointestinal SMCs such as delayed rectifier fast activating α-dendrotoxin (α-DTX)-sensitive K^+^ channels (Kv) [13,14,15,16], large-conductance Ca^2+^-activated K^+^ channels (BK) [17,18] and small conductance Ca^2+^-activated K^+^ channels (SK) [19,20].

ADPN exerts its effects mainly through the activation of two transmembrane receptors, AdipoR1 and AdipoR2, whose expression has been revealed in the brain as well as in a variety of mammalian peripheral tissues [21], including the gastrointestinal tract [5,22]. The biological effects of ADPN are related to the expression of tissue-specific receptors and to the different signaling pathways engaged. In this regard, in the vascular system, the hormone initiates the AMP-activated protein kinase (AMPK)-mediated nitric oxide synthase (NOS) activation, leading to an increase of nitric oxide (NO) production [23]. Notably, AMPK, an enzyme characterized by a tissue-specific mechanism of activation [24], acts at central level (hypothalamus) to control appetite and body weight, making it an important pharmacologic target for the potential treatment of obesity and eating disorders [25,26].

In addition to the central anorexigenic effect of ADPN [27], a peripheral mechanism that could strengthen such action can be represented right by the stomach wall distension, which is a well-known satiety signal, favored by the gastric smooth muscle cell relaxation induced by the hormone. In this view, it could be important to elucidate the signaling pathways involved in the effects of ADPN at the gastrointestinal level, but no data are present in the literature. Accordingly, the aim of this study was to investigate the possible mechanisms through which ADPN exerts its inhibitory effects at the gastric level.

## 2. Results

### 2.1. L-NNA Prevents the Mechanical Inhibitory Effect of ADPN on the Gastric Fundus

**Figure 1:**
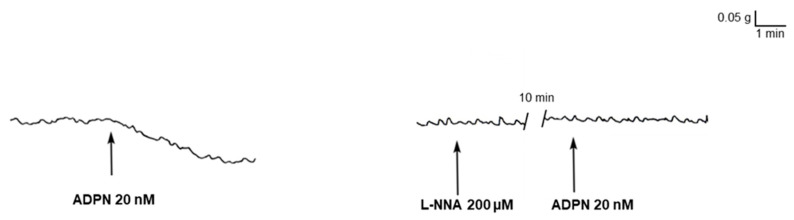
Lack of effects of adiponectin (ADPN) on carbachol (CCh)-precontracted gastric strips in the presence of L-N^G^-nitro arginine (L-NNA). Typical tracing (**left** hand panel) showing the long-lasting and slight (note the magnitude of the amplification) decay of the strip basal tension caused by 20 nM ADPN. Such an effect, already appreciable within 1 min of contact time, persists up to 30 min (longer time not observed). When ADPN is added to the bath medium 15 min after the NO synthesis inhibitor L-NNA (200 μM), it is no longer able to induce the decay of the strip basal tension (**right** hand panel). Traces obtained from two different preparations.

At basal tension and in the presence of guanethidine, the addition of carbachol (CCh, 1 μM) to the bath medium (*n* = 8 strips) elicited a rapidly arising contraction (mean amplitude 1.2 g ± 0.2 g), which persisted until washout. As previously observed in CCh-precontracted strips [5], 20 nM ADPN caused, after 1–2 min of contact time, a progressive and long-lasting decay of the basal tension (mean amplitude 0.16 g ± 0.03 g, *n* = 8) (Figure 1) which was not affected by tetrodotoxin (TTX, 1 µM) (*p* > 0.05, *n* = 2), thus indicating that the hormone action occurs on the smooth muscle. The chosen dose of ADPN (20 nM) is that previously reported to be effective in gastric preparations [5,6].

In the presence of the NO synthesis inhibitor, L-N^G^-nitro arginine (L-NNA, 200 μM), the addition of 20 nM ADPN to the bath medium (*n* = 6 strips) had no longer influence on the strip basal tension (Figure 1) (*p* > 0.05), suggesting that the effects of the hormone are mediated by NO. 

Based on the present observations, we first performed electrophysiological experiments to investigate the role of NO in the effects of ADPN on the gastric SMCs.

### 2.2. L-NNA Hampers the Effects of ADPN on the Resting Membrane Potential and on the Membrane Passive Properties of SMCs

To better investigate the effect of ADPN in the presence of L-NNA observed in the mechanical experiments, we firstly conducted electrophysiological experiments on the resting membrane potential (RMP) of SMCs.

**Figure 2:**
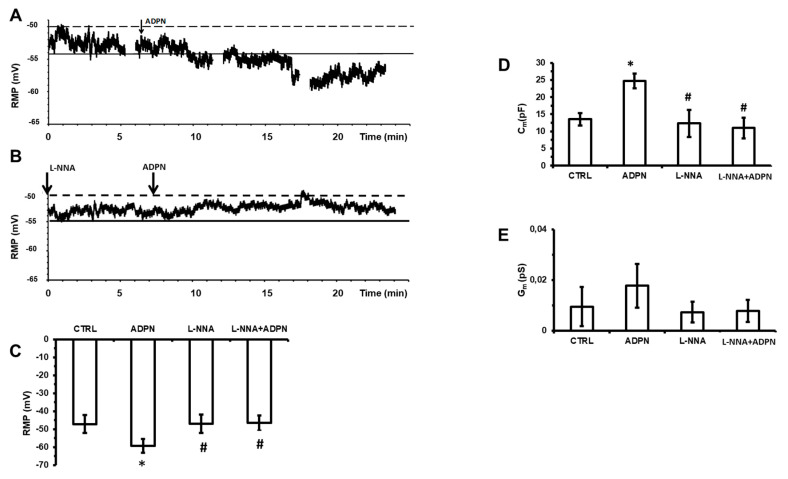
The effects of ADPN on the resting membrane potential and on passive membrane properties of a gastric smooth muscle cell (SMC) are prevented by L-NNA. (**A**) Typical tracing of resting membrane potential (RMP) from a single gastric SMC. Asynchronous and irregular waves of resting membrane potential recorded in basal condition and almost 15 min after ADPN (20 nM) addition (arrow). ADPN induced a persistent hyperpolarization occurring right after about 2–3 min from its application. (**B**) Typical tracing of RMP recorded in the presence of L-NNA (arrow) and almost 15 min after ADPN (20 nM) addition (arrow). (**A**,**B**) Continuous line is the mean value of resting membrane potential and dashed line is the maximal value of the peak waves. (**C**) Bar charts representing the effects of ADPN alone or in its concomitant presence of L-NNA on the RMP. (**D**) Bar charts showing the effect of ADPN and ADPN+L-NNA on the cell capacitance (Cm), index of cell surface, of the SMCs. (**E**) Bar charts showing the effect of ADPN and ADPN+L-NNA on the membrane conductance (Gm), index of membrane permeability, of the SMCs. For any parameter, data are evaluated in control condition (CTRL), 15 min after adiponectin addition to the bath solution (ADPN) and in the concomitant presence of L-NNA and ADPN (L-NNA+ADPN). One-way ANOVA with Bonferroni’s post hoc test was used for multiple comparisons. *, *p* < 0.05 vs. control; #, *p* < 0.05 vs. ADPN. Values are means ± SD. Mean values are listed in Table 1.

The acute addition of ADPN to the bath solution caused a statistically significant hyperpolarization (Figure 2A,C, Table 1), which is quite in accordance with the decay of the basal tension observed in the mechanical experiments. Such hyperpolarization was already appreciable 1–3 min following the hormone application and reached the most negative (hyperpolarized) value after about 15 min (Figure 2A). In the presence of L-NNA (Figure 2B,C, Table 1), ADPN was no longer able to induce any hyperpolarization, suggesting the NO involvement in the alteration of membrane potential.

Then, the effect of the hormone in the presence of L-NNA was investigated also on the passive membrane properties of the SMC. As observed in our preliminary report [6], ADPN (20 nM) induced a significant increase of the cell capacitance (Cm) compared to the control, whereas the membrane conductance (Gm) was reduced. Notably, all of the effects of ADPN on Cm (Figure 2D, Table 1) and Gm (Figure 2E, Table 1) were prevented by L-NNA. These data confirm that ADPN was able to affect the SMC membrane properties, suggesting that the remodeling of the cell surface and of its resting permeability involve the NO pathway. 

### 2.3. Effects of ADPN on the Ion Currents in the Presence of L-NNA

#### 2.3.1. Effects of ADPN on the Different Types of Voltage-Dependent Outward K^+^ Currents

**Figure 3:**
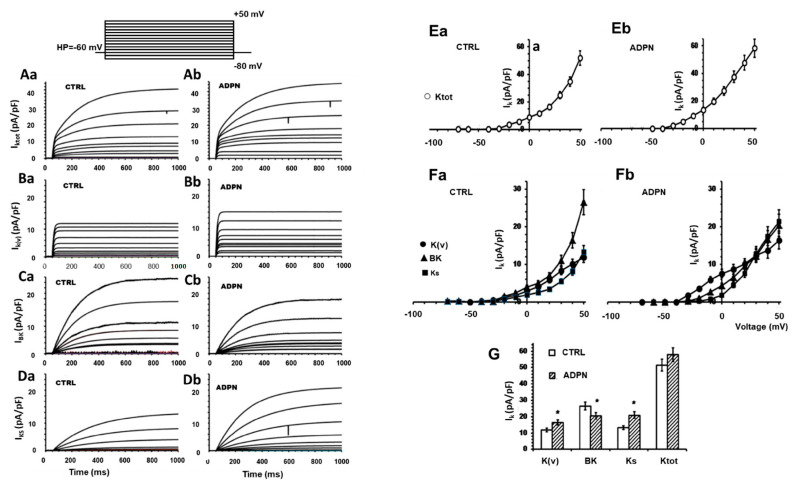
Effects of ADPN on the different voltage-dependent K^+^ currents in an SMC from the gastric fundus. (**Aa**,**Ab**)Total outward K^+^ currents traces, elicited by voltage steps from −80 to +50 mV (holding potential (HP) = -60 mV) in nifedipine (10 μM) containing solution, in control condition (**Aa**), and in the presence of ADPN 20 nM (**Ab**). Current values are normalized to cell capacitance. The pharmacological dissection allowed us to systematically distinguish three kinds of K+ currents: IKv (**Ba**,**Bb**); IBK (**Ca**,**Cb**); and IKs (**Da**,**Db**). Current values are normalized to cell capacitance. (**Ea**,**Eb**) I–V plots related to IK,TOT in CTRL (**Ea**) and in the presence of ADPN (**Eb**). (**Fa**,**Eb**) I–V plots related to IKV, IBK, and IKs, in CTRL conditions (**Fa**) and in the presence of ADPN (Fb). The continuous lines through the experimental data represent the fitted Boltzmann function. Current values are normalized to cell capacitance. Statistical significance is not depicted in the figure for clarity. (**G**) Bar charts representing the amplitude of K+ current for IK,TOT, IKV, IBK, and IKs, in CTRL conditions and in the presence of ADPN. Comparison of the maximal current values recorded at +50 mV; * *p* < 0.05 of ADPN vs. CTRL. All of the data are mean values ± SEM. In each experimental condition, data are from CTRL n = 30 cells, ADPN n = 9 cells (5 mice).

First of all, we evaluated the effects of ADPN on K^+^ channels of the SMCs, which are supposed to be mostly implicated in the regulation of the RMP. The acute addition of ADPN was able to cause an increase of I_K_ compared to control, as observed also in our preliminary study [6]. To clarify the different types of the K^+^ channel mostly involved in the effects of the hormone, we here operated a pharmacological dissection of the overall I_K,TOT_, according to the previously published procedure [28]. I_K,TOT_ (Figure 3A) was elicited by a voltage pulse protocol of stimulation in the modified bath control solution containing BaCl_2_, 4-aminopyridine (4-AP), TTX, nifedipine and heptanol (see Materials and Methods). By this system, we were consistently able to distinguish, in control condition, the fast activating α-dendrotoxin (α-DTX)-sensitive I_Kv,_ the large conductance Ca^2+^-activated K^+^ current that was Iberiotoxin (IbTx) sensitive, namely BK current (I_BK_), and the slowly activating chromanol (Chr)-sensitive I_Ks_ (Figure 3(Ba,Bb–Da,Db)). After ADPN addition, we could clearly observe an increase in I_Kv_ and I_Ks_ amplitudes (Figure 3(Ba,Bd,Da,Db)). Thus, we can assess that the outward K^+^ current increase induced by ADPN was mostly due to the potentiation of the K_v_ and K_s_ currents. In contrast, BK resulted reduced by ADPN treatment (Figure 3(Ca,Cb)).

To better evaluate the general trend of this event, we calculated the I-V relationship related to all of the experiments done, reporting the normalized mean current maximal amplitude for any voltage step applied (Figure 3(Ea,Eb,Fa,Fb)). The analysis of the I-V plot confirmed that ADPN treatment enhanced the fast and low-threshold activated I_Kv_ and the slow and high-threshold activated I_Ks_, thus enabling the compensation of the fast and slow changes of the resting membrane potential. In contrast, the Ca^2+^-dependent K^+^ currents with an intermediate voltage threshold was mainly reduced. The voltage threshold of activation of the different currents was −40 ± 5, −30 ± 5 and −20 ± 4 mV for I_Kv_, I_BK_, and I_Ks_, respectively (Figure 3G). However, in a lower percentage of cells (less than 8%), we observed an increase in I_K,TOT_ current due to BK (not shown) as also reported for vascular tissues [29]. Thus, we can conclude that in gastric SMC, ADPN increases the outward K^+^ current, mainly enhancing Kv and K_s_ currents. 

#### 2.3.2. Effects of ADPN on the Different Types of Voltage-Dependent Inward Ca^2+^ Current

**Figure 4:**
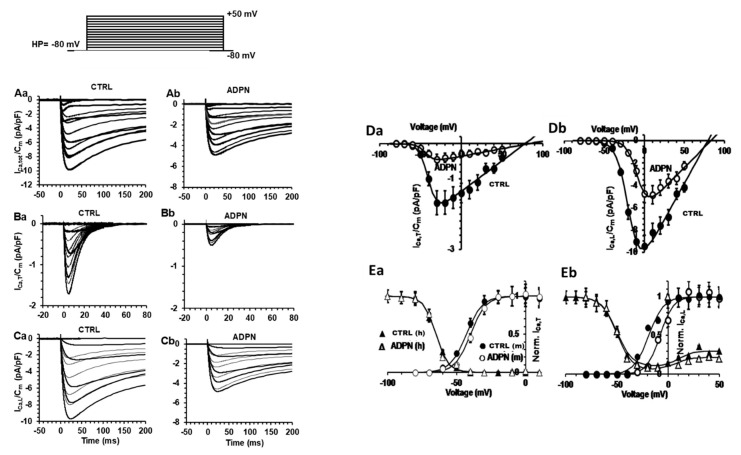
Effects of ADPN on the voltage-dependence of T- and L-type Ca^2+^ channels activation. (**Aa**,**Ab**) Current traces of total inward Ca^2+^ currents (I_Ca_) recorded in control SMC (**Aa**) and in the presence of ADPN (**Ab**). Current values are normalized to cell capacitance. (**Ba**,**Bb**) Representative time course of T-type Ca^2+^ currents (I_Ca,T_) recorded in the presence of nifedipine from a control cell (**Ba**) and after ADPN addition (**Bb**). (**Ca**,**Cb**) Time course showing L-type Ca^2+^ currents (I_Ca,L_) recorded in the high TEA-Ca^2+^ solution from a control cell (**Ca**) and after ADPN addition (**Cb**). Only the first 200 ms of the pulse are depicted. (**Da**,**Db**) I-V plots related to I_Ca,T_ (**Da**) and I_Ca,L_ (**Db**) in control (CTRL, filled circles) and ADPN-treated (ADPN, open circles) SMCs from the gastric fundus. Data are normalized for the mean cell capacitance. The lines through the experimental data are the fit with a Boltzmann function. (**Ea**,**Eb**) Steady-state activation and inactivation analysis for I_Ca,T_ (**Ea**) and I_Ca,L_ (**Eb**). Effect of ADPN on I_CaT_ and I_Ca,L_ activation (open circles, m) with respect to control (filled circles, m); lack of effects on inactivation (CTRL, filled triangles, h; ADPN, open triangles, h). Note the U-shaped inactivation curve at positive potentials for I_Ca,L_ that is depressed in ADPN-treated cells. Current values are normalized to cell capacitance. All data are mean values ±SEM Statistical significance is not depicted in the figure for clarity but is reported for the various Boltzmann parameters in Table 2. Data are from CTRL *n* = 20 cells, ADPN *n* = 7 cells, (5 mice).

As already observed by Idrizaj et al., 2019 [6], ADPN also influences calcium channels by inducing a reduction of I_Ca_ amplitude. In the present study, we intended to clarify if ADPN could affect a specific type of Ca^2+^ current that is, T- and/or L-type Ca^2+^ currents (I_Ca,T_ and I_Ca,L_). Thus, we applied a suitable pulse protocol of stimulation starting from a holding potential (HP) = −80 mV for evoking the Ca^2+^ currents using the high-tetraethylammonium (TEA) solution in the bath (see Materials and Methods section). When this type of experiment was performed in the presence of nifedipine (10 µM) to block L-type Ca^2+^ channels, we could observe a residual low voltage activated current with a rapid and transient time course (peak time, t_p_ = 5.5 ± 0.5 ms), suggesting the occurrence of I_Ca,T_ (Figure 4(Ba)). The addition of ADPN decreased the maximal size of I_Ca,T_ slightly affecting its time to peak (t_p_ = 5.8 ± 0.5 ms) and negatively shifting the maximal activation from −25 to −30 mV (Figure 4(Bb)), Table 2). By subtracting the current traces obtained in the presence of nifedipine from the total currents (data not shown), we obtained the L-type Ca^2+^ channel contribution alone (Figure 4(Ca)): we observed high-voltage-activated current traces with a slow decay and with the maximum amplitude reached in 22.7 ± 2 ms evoked by a 0-mV step pulse. Once again, the addition of ADPN decreased the size of I_Ca,L_ (Figure 4(Cb)) apparently without altering its time to peak (Table 2), but shifting the voltage value of the pulse required to evoke the maximal current amplitude from 0 to 10 mV (Figure 4(Da,Db)). 

To better evaluate the general behavior of the phenomenon, we calculated the I-V relationship related to all the experiments done, reporting the normalized mean I_Ca_ maximal amplitude for any voltage step applied (Figure 4(Da,Db)). The evaluation of the current voltage dependence was performed on I_Ca,T_ and I_Ca,L_ separately in control conditions and under ADPN treatment. Any data point of the plot is the mean value ± SEM of the peak amplitudes obtained for any voltage step applied in all of the experiments done. Control I-V curves related to I_Ca,T_ and I_Ca,L_ recorded from SMCs of gastric fundus are shown in Figure 4(Da,Db), and the line through the filled symbols represents the fit of a Boltzmann function. Thus, the present experiments show that the reduction of I_Ca_ amplitude caused by ADPN addition concerns both the types of current. However, ADPN did not affect the voltage dependence of inactivation neither of T- nor L-type Ca^2+^ current (Figure 4(Ea,Eb)). ADPN positively shifted the half-maximal activation voltage value of I_Ca,T_ activation of 5.1 ± 1 mV and that of I_Ca,L_ of 19.8 ± 4 mV compared to control. The related Boltzmann parameters with statistical significance are listed in Table 2.

Moreover, it is to note that the inactivation curves did not steadily keep on at zero level at positive potentials but progressively increased causing a sort of U-shaped inactivation curve. This behavior may suggest that inactivation was Ca^2+^-dependent. ADPN depressed this phase, which was probably due to the minor intracellular [Ca^2+^]_i_ as a consequence of the decreased Ca^2+^ influx through L-type Ca^2+^ channels.

#### 2.3.3. L-NNA Prevents the Effect of ADPN on K^+^ Channels but Not on ca^2+^ Channels

**Figure 5:**
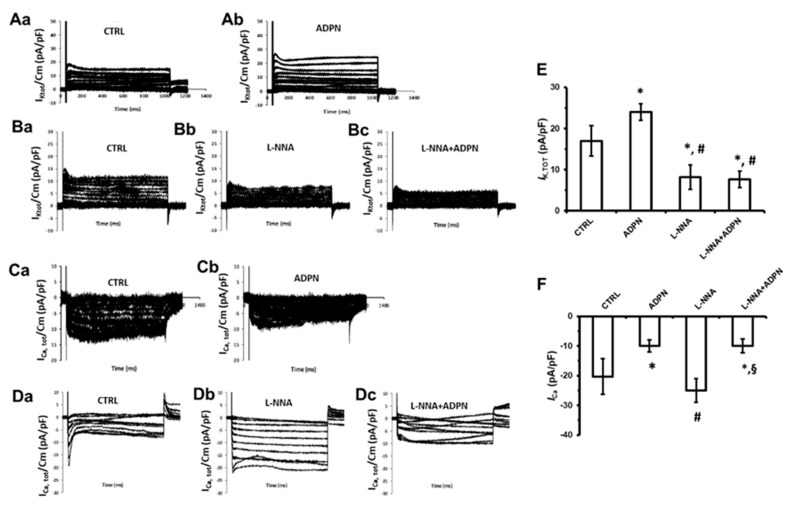
Effects of ADPN on the ion currents in a single SMC in the presence of L-NNA. (**Aa**,**Ab**) Typical tracing of total outward K^+^ current recorded in a single control cell (**Aa**) and after ADPN addition (**Ab**). (**Ba–Bc**) Typical tracing of total outward K^+^ current recorded in a single control cell (**Ba**), in L-NNA (**Bb**), and after ADPN addition in the presence of L-NNA (**Bc**). (**Ca**,**Cb**) Typical tracing of total inward Ca^2+^ current recorded in a single control cell (**Ca**) and after ADPN addition (**Cb**). (**Da–Dc**) Typical tracing of total inward Ca^2+^ current recorded in a single control cell (**Da**), in L-NNA (**Db**), and after ADPN addition in the presence of L-NNA (**Dc**). All current values are normalized to cell capacitance. (**E**) Bar charts representing the effects of ADPN, L-NNA, and L-NNA+ADPN on the total K^+^ current outflow. (**F**) Bar charts representing the effects of ADPN, L-NNA, and L-NNA+ADPN on the total Ca^2+^ current inflow. Bar charts are related to the current amplitude evoked by the +50 mV step pulse, measured at the end of the stimulus for K^+^ current and at the peak amplitude for Ca^2+^ currents (Ip, peak current). Data evaluated in control condition (CTRL), 15 min after adiponectin addition to the bath solution (ADPN) and after adding adiponectin in the presence of L-NNA (L-NNA+ADPN). (**E**,**F**) One-way ANOVA with Bonferroni’s post hoc test was used for multiple comparisons. *, *p* < 0.05 vs. control; **#**, *p* < 0.05 vs. ADPN; **§**, *p* < 0.05 vs. L-NNA. Values are means ± SD. Current mean values are listed in Table 1.

To elucidate the whole scenario, we tested the effect of ADPN when the NO synthesis was inhibited also on the occurrence of ion currents I_K_ and I_Ca_. In the present research, we observed that the hormone was no more able to induce the increase of the total K^+^ current outflow in the presence of L-NNA (Figure 5(Ba–Bc),E, Table 1). Typical tracings are shown in Figure 5(Aa,Ab–Da–Dc) for the different conditions. Interestingly, ADPN in the presence of L-NNA continued to reduce Ca^2+^ entry, especially the early transient phase of the trace with a rapid transient time course (Figure 5(Da–Dc),F, Table 1). Such observation may suggest that, in addition to NO, other signaling effectors could be involved in the mechanism by which ADPN modulates Ca^2+^ channels in gastric smooth muscle. 

### 2.4. Dorsomorphin Hampers the Effects of ADPN on the Electrophysiological Properties and Ion Currents of SMCs

Based on the above observations, we decided to test the involvement of AMP-activated protein kinase (AMPK) that is usually recruited upon AdipoR1 activation [30,31,32,33] and is known to mediate NOS activation, leading to an increase of NO production [34,35,36].

**Figure 6:**
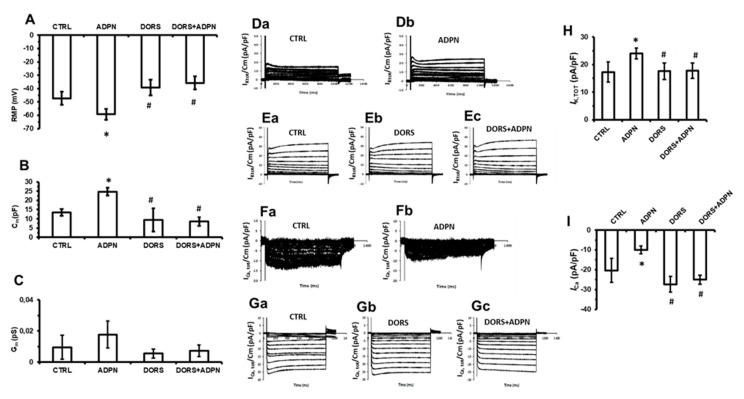
Lack of effects of ADPN on SMC electrophysiological properties and ion currents in the presence of dorsomorphin (DORS). (**A**) Bar charts showing the effect of ADPN and DORS+ADPN on the resting membrane potential (RMP) of the SMCs. (**B**) Bar charts showing the effect of ADPN and DORS+ADPN on the cell capacitance (Cm), the index of cell surface, of the SMCs. (**C**) Bar charts showing the effect of ADPN and DORS+ADPN on the membrane conductance (Gm), the index of membrane permeability, of the SMCs. (**Da**,**Db**) Typical tracing of total outward K^+^ current recorded in a single control cell (**Da**) and after ADPN addition (**Db**). (**Ea–Ec**) Typical tracing of total outward K^+^ current recorded in a single control cell (**Ea**), in dorsomorphin (**Eb**) and after ADPN addition in the presence of dorsomorphin (**Ec**). (**Fa**,**Fb**) Typical tracing of total inward Ca^2+^ current recorded in a single control cell (**Fa**) and after ADPN addition (**Fb**). (**Ga–Gc**) Typical tracing of total inward Ca^2+^ current recorded in a single control cell (**Ga**), in dorsomorphin (**Gb**) and after ADPN addition in the presence of dorsomorphin (**Gc**). All current values are normalized to cell capacitance. (**H**) Bar charts representing the effects of ADPN, DORS, and DORS+ADPN on the total K^+^ current outflow. (**I**) Bar charts representing the effects of ADPN, DORS, and DORS+ADPN on the total calcium current inflow. Bar charts are related to the current amplitude evoked by the +50 mV step pulse, measured at the end of the stimulus for K^+^ current and in the initial part of the stimulus for Ca^2+^ currents (Ip, peak current). Data evaluated in control condition (CTRL), 15 min after adiponectin addition to the bath solution (ADPN), and after adding adiponectin in the presence of dorsomorphin. One-way ANOVA with Bonferroni’s post hoc test was used for multiple comparisons. *, *p* < 0.05 vs. control; #, *p* < 0.05 vs. ADPN. Current mean values ±SD are listed in Table 1.

In the presence of dorsomorphin (10 µM), which was used to block upstream the AMPK pathway, ADPN was no more able to cause significant alterations of RMP, Cm, or Gm (Figure 6A–C, Table 1). As well, adding ADPN in the presence of dorsomorphin did not alter K^+^ (Figure 6(Ea–Ec)) or Ca^2+^ current amplitude compared to control (Figure 6(Ga–Gc)). Typical tracings are shown in Figure 6(Da,Db–Ga–Gc) for the different conditions. Data were not statistically different compared to control, indicating that ADPN was ineffective in the presence of the AMPK inhibitor.

### 2.5. ADPN Increases AMPK Signaling

**Figure 7:**
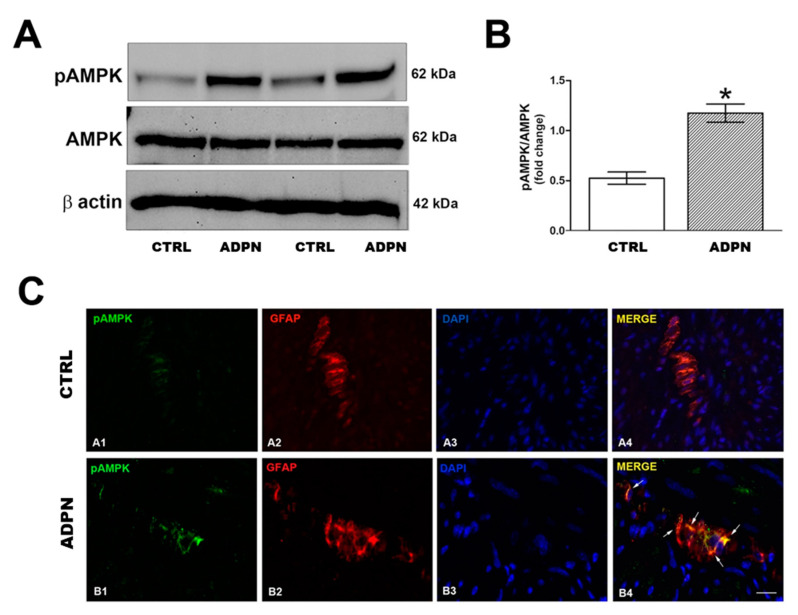
Involvement of AMPK signaling in ADPN action. (**A**) Effect of ADPN on AMPK signaling pathway in mouse gastric fundus assayed by Western blotting: representative bands from a typical experiment. (**B**) quantitative analysis. Columns are means ± SEM. Significance of differences (Student’s *t*-test for two independent samples): * *p* < 0.05 vs. controls (CTRL). (**C**) Representative photomicrographs of gastric tissue from control and ADPN-treated mice showing double immunofluorescence labeling. (**A1–A4**) co-localization of phosphoAMPK (pAMPK) and GFAP in control mice: (**A1**) pAMPK signal (green channel); (**A2**) GFAP signal (red channel); (**A3**) DAPI; (**A4**) merged images. (**B1–B4**) co-localization of pAMPK and GFAP in ADPN-treated mice: B1 pAMPK signal (green channel); (**B2**) GFAP signal (red channel); (**B3**) DAPI; (**B4**) merged images. Scale bar: 10 µm.

To confirm that ADPN action on gastric preparation involves AMPK activation, we performed Western blotting analysis. We found that ADPN treatment activated the AMPK signaling in the mouse gastric fundus as demonstrated by the significant increase of phosphoAMPK (pAMPK) expression compared to the control samples (Figure 7A). The results obtained by Western blotting were confirmed by immunofluorescence analysis: pAMPK immunostaining appeared increased in the gastric samples from ADPN-treated mice as compared to the controls (Figure 7B), while no difference was detected on AMPK expression (data not shown). Specifically, ADPN induced activation of the AMPK pathway in the glial cells of myenteric plexus as shown by the co-localization of pAMPK and GFAP immunostaining (Figure 7B).

### 2.6. AdipoR1 Detection

**Figure 8:**
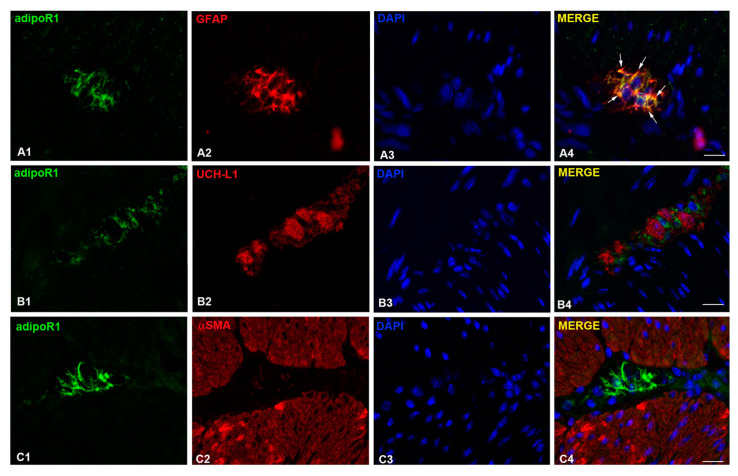
Localization of AdipoR1 in the gastric fundus. Representative photomicrographs of gastric tissue showing double immunofluorescence labeling. (**A1–A4**) Co-localization of adipoR1 and GFAP: (**A1**) adipoR1 signal (green channel); (**A2**) GFAP signal (red channel); (**A3**) DAPI; (**A4**) merged images. (**B1–B4**) Co-localization of adipoR1 and UCH-L1: (**B1**) adipoR1 signal (green channel); (**B2**) UCH-L1signal (red channel); (**B3**) DAPI; (**B4**) merged images. (**C1–C4**) Co-localization of adipoR1 and αSMA; (**C1**) adipoR1 signal (green channel); (**C2**) αSMA signal (red channel); (**C3**) DAPI; (**C4**) merged images. Scale Bars: 10 µm.

Several studies indicate that AMPK is usually recruited under AdipoR1 activation [30,31,32,33]. Accordingly, we investigated the presence of this receptor in the murine gastric fundus specimen, by performing a dual immunofluorescence labeling. The results obtained revealed that AdipoR1 was expressed by glial cells associated with neurons in the myenteric plexus (Figure 8). In fact, the co-localization of adipoR1 and GFAP immunoreactivities was observed within the myenteric plexus (Figure 8(A1–A4)), as showed by the resulting yellow color in the merged image (Figure 8(A4), white arrows), while no co-localization appeared between adipoR1 and the neuronal marker UCH-L1 (Figure 8(B4)). No adipoR1 signal was clearly detected in the gastric smooth musculature (Figure 8(C1–C4)).

## 3. Discussion

The present study indicates that ADPN exerts its inhibitory action at the gastric smooth muscle level likely acting on AdipoR1 and involving the AMPK/NO pathway.

In addition to the previously reported neuromodulatory action of ADPN on the mechanical responses of the mouse gastric fundus [5], we here observed that the hormone exerts its effects on the smooth muscle. The ability of the NO synthesis inhibitor, L-NNA, to abolish the decay of the basal tension caused by ADPN on gastric strips indicates the involvement of the NO pathway in such effects. Accordingly, NO appears to be a shared target pathway in the hormonal control of gastrointestinal motility [5,37,38], and the ability of ADPN to increase NO synthase expression has been reported in different smooth muscle tissues as in the vascular one [34,39]. In agreement, in the present electrophysiological experiments, we observed that all the effects of ADPN on the membrane passive properties (membrane capacitance and conductance) of gastric SMCs were prevented by L-NNA, underlying the involvement of NO. Moreover, the hyperpolarization induced by ADPN, which is quite in accordance with the decay of the basal tension observed in the mechanical experiments, was also prevented by the NO synthesis inhibitor L-NNA. However, in the present experiments, we observed that in the presence L-NNA, ADPN was no longer able to increase the K^+^ current outflow, but it still reduced Ca^2+^ entry. Such observations strongly indicate that, in addition to NO, other signaling effectors should be involved in the mechanism of action of ADPN on the gastric muscle. In this regard, ADPN has been reported to engage several signaling paths in other smooth muscles [40,41]. Among them, AMP-activated protein kinase (AMPK) frequently mediates the effect of the hormone in different organs and tissue [42,43,44,45]. Accordingly, in our murine gastric fundus preparations, all the effects of ADPN on RMP and membrane passive proprieties of SMC were prevented by blocking upstream with the inhibitor of AMPK dorsomorphin. This was quite an expected result, since previous papers reported the ability of the hormone to initiate AMPK-mediated NOS activation, thus leading to an increase of NO production [34,35,36]. Interestingly, in the presence of dorsomorphin, the hormone could not induce any of its effect either on K^+^ or Ca^2+^ current. This observation strongly points out AMPK involvement in the mechanism of action of ADPN. Indeed, our results indicate that ADPN leads to an enhanced K^+^ outflow by increasing NO production through AMPK activation. In contrast, the reduction of Ca^2+^ influx is probably due to the AMPK-induced activation of other signaling effectors different from NO. Further studies are necessary to better investigate on the other possible signaling paths activated by AMPK, through which ADPN may modulate Ca^2+^ channels on the gastric smooth muscle.

**Figure 9:**
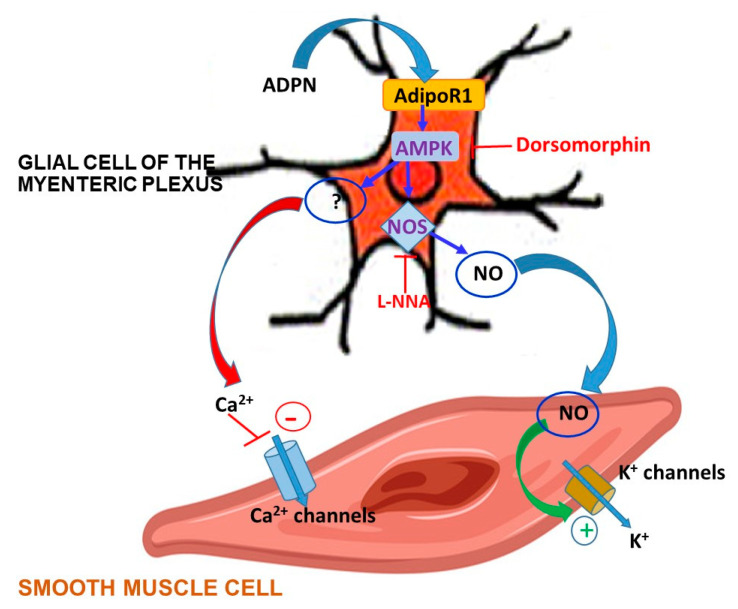
Schematic model depicting the putative action of ADPN on a glial cell of the myenteric plexus influencing SMC ion channels. ADPN interacts with AdipoR1 located on enteric glial cell membrane activating the AMPK pathway. This in turn promotes NOS activity, which leads to NO production. NO, as a gaseous molecule, spreads to the nearby SMC, ultimately affecting its bioelectric properties. Particularly, K^+^ channel activation results enhanced (+), whereas the Ca^2+^ channel activation results decreased (-) by ADPN. However, the modulation of Ca^2+^ channels may not be due only to NO but can involve further downstream effectors to AMPK (as indicated by the question mark). The blockade of AMPK by dorsomorphin and the inhibition of NO synthesis by L-NNA are also illustrated in the scheme.

The involvement of the AMPK pathway in the mechanism of action of ADPN is further supported by the Western blotting and immunofluorescence results: ADPN treatment significantly increased pAMPK expression in the glial cells associated with neurons in the myenteric plexus of the mouse gastric fundus. Moreover, the presence of AdipoR1 in our specimens, prevalently expressed by glial cells in the myenteric plexus, is in keeping with the involvement of the AMPK pathway, since its recruitment is largely proven in this receptor activation [30,31,32,33]. Based on our overall results, we therefore propose that ADPN, activating AdipoR1 on the glial cells, leads to NO production via the AMPK/NOS axis. NO, as a gaseous molecule, can easily diffuse from glial to nearby smooth muscle cells, so influencing their electrophysiological properties. Accordingly, enteric glial cells have been reported to play an important role in the regulation of gastrointestinal motility [46], even if the mechanisms behind this effect need to be elucidated. A representative summary scheme of our hypothesis based on the present results is shown in the Figure 9. Our research designates AMPK as a key mediator of ADPN effects at the gastric level, offering the first direct evidence that the hormone exerts its inhibitory effects, mainly through the AMPK-NO pathway leading to muscle relaxation. The ability of ADPN to induce muscle relaxation leads to an increased gastric wall distension. From a physiological point of view, this can represent an additional peripheral satiety signal [47]. In fact, gastric motor changes represent well-known peripheral factors that are able to send signals to the central structures involved in the regulation of food intake [48]. Thus, we speculated that the here-observed peripheral inhibitory effects induced by ADPN through the activation of AMPK are part of a control system aimed to regulate food intake, which might concur to suppress feeding behavior. Accordingly, acting at the central level (hypothalamus), AMPK controls appetite and body weight [26], making it an important pharmacologic target for the potential treatment of obesity and eating disorders. In this study, we observed the involvement of this path for the first time at the peripheral level in gastric preparations, suggesting that satiety signals originating in diverse regions actually engage the same effectors, strengthening a common aim. 

In conclusion, our results support the physiological relevance of ADPN as a part of the multifarious hormonal circuits that coordinate energy homeostasis and food intake, acting at central and peripheral levels. These observations could provide novel bases for the opening of new considerations in the search of a possible therapeutic treatment for obesity and/or eating disorders.

## 4. Materials and Methods 

### 4.1. Ethical Approval

The experimental protocol was designed in compliance with the guidelines of the European Communities Council Directive 2010/63/UE and the recommendations for the care and use of laboratory animals approved by the Animal Care Committee of the University of Florence, Italy, with authorization from the Italian Ministry of Health nr. 787/2016-PR.

### 4.2. Animals

Experiments were performed on 8- to 12-week-old female mice (C57BL/6; Charles River, Lecco, Italy). The mice were fed standard laboratory chow and water and were housed under a 12-h light/12-h dark photoperiod and controlled temperature (21 ± 1 °C). The mice were killed by prompt cervical dislocation to minimize animal suffering.

### 4.3. Mechanical Experiments

A previously reported [5,38] full-thickness longitudinal strips were dissected from the stomach and mounted in 5-mL organ baths containing Krebs–Henseleit solution composed of 118 mM NaCl, 4.7 mM KCl, 1.2 mM MgSO_4_, 1.2 mM KH_2_PO_4_, 25 mM NaHCO_3_, 2.5 mM CaCl_2_, and 10 mM glucose (pH 7.4) and bubbled with 95% O_2_-5% CO_2_. The temperature was maintained within a range of 37 ± 0.5°C. One end of each preparation was tied to a platinum rod, and the other was connected to a force displacement transducer (FT03; Grass Instruments, Quincy, MA, USA) by a silk thread for continuous recording of isometric tension. The transducer was coupled to a polygraph (7 K; Grass Instruments). Preparations were allowed to equilibrate for 1 h under an initial load of 0.8 g. During this period, the preparations underwent repeated and prolonged washes with Krebs–Henseleit solution to prevent accumulation of metabolites in the organ baths.

All functional experiments were carried out in the presence of 1 µM guanethidine sulfate and 1 µM carbachol (CCh) to rule out the adrenergic and the cholinergic influences, respectively. When contraction elicited by CCh reached a stable plateau phase, ADPN or drugs were applied. The interval between two subsequent applications of CCh was no less than 30 min, during which repeated and prolonged washes of the preparations with Krebs–Henseleit solution were performed. The effects of ADPN (20 nM) were investigated alone as well as 15 min following the addition of TTX or L-NG-nitro arginine (L-NNA) to the bath medium. 

### 4.4. Electrophysiological Experiments

For electrophysiological recordings, the stomach was quickly removed and cleaned with Krebs–Henseleit solution as reported previously [5,6], which consisted of 118 mM NaCl, 4.7 mM KCl, 1.2 mM MgSO_4_, 1.2 mM KH_2_PO_4_, 25 mM NaHCO_3_, 2.5 mM CaCl_2_, and 10 mM glucose (pH 7.4). At least two strips of gastric fundus were obtained from each stomach. A full-thickness strip was cut and pinned to a Sylgard (Dow Corning, Midland, MI, USA)-coated dissecting Petri dish filled with Krebs–Henseleit solution. First, we pinned the mucosal side up to dissect carefully the mucosa and submucosa away under a dissecting microscope. The residual tissue was re-pinned serosal side up, and the connective tissue was removed in order to expose the smooth muscle layer. The obtained tissue was finally pinned, serosal side up, in the recording chamber with a Sylgard floor. During the experiments, the tissue was continuously superfused (Pump 33, Harvard Apparatus, Holliston, MA, 01746, United States) at a rate of 1.8 mL min-1 with Krebs–Henseleit solution. Intracellular recording was made by a conventional high resistance glass microelectrode [28] inserted in a cell of the longitudinal smooth muscle layer (SMC). Microelectrodes were obtained by using a micropipette vertical puller (Narishige PC-10) from borosilicate glass (GC 100-7.5; Clark, Reading, UK) and were normally filled with the following internal solution (mM): KCl 130, NaH_2_PO_4_ 10, CaCl_2_ 0.2, ethylene-bis(oxyethylenenitrilo) tetraacetic acid (EGTA) 1, MgATP 5 and 4-(2-hydroxyethyl)-1-piperazineethanesulfonic acid (HEPES)/KOH 10. Once filled, the pipette resistance measured 60–70 MΩ. The pH was set to 7.4 with NaOH and to 7.2 with tetraethylammonium hydroxide (TEA-OH) for bath and pipette solution, respectively. For RMP recording in current-clamp experiments, we used Krebs–Henseleit as the control bath solution. The delayed rectifier K^+^ current records were achieved in voltage-clamp condition using a modified Krebs–Henseleit solution with different specific channels blockers added: BaCl_2_ to block the inward rectifier K^+^ current, I_Kir_, nifedipine to block L-type Ca^2+^ current, TTX to block Na^+^ current, 4-aminopyridine (4-AP) to block transient outward K^+^ current (I_to_). The type of voltage-dependent delayed rectifier current involved in the total outward K^+^ records was evaluated by pharmacological dissection [28,49,50]. Accordingly, three types of delayed rectifier currents, I_BK_, I_Ks_, and I_Kv_ were identified by the following specific blockers: iberiotoxin (IbTx), chromanol (Chr) and α-dendrotoxin (α-DTX), respectively. 

To record only Ca^2+^ currents, the microelectrodes were filled with the following filling electrode solution (mM): 150 CsBr, 5 MgCl_2_, 10 ethylene-bis(oxyethylenenitrilo) tetraacetic acid (EGTA), 10 (4-(2-hydroxyethyl)-1-piperazineethanesulfonic acid) (HEPES). Moreover, we used a Na^+^- and K^+^-free high-TEA external solution with the following composition (mM): 10 CaCl_2_, 145 TEABr and 10 HEPES. Nifedipine was used to test I_Ca,L_ occurrence.

The gap junctional blocker heptanol (1 mM) was added to any bath solution to avoid electrical coupling among the SMCs and to enable the recording of any phenomena elicited only from the impaled cell [6,49,51,52]. 

Any current amplitude was normalized to cell linear capacitance Cm to consent the evaluation of test current recorded from cells of different size; in fact, Cm is usually considered an index of cell-surface area, presuming that the membrane-specific capacitance has a constant value of 1 µF/cm^2^. 

#### Pulse Protocols of Stimulation

We recorded the RMP of the SMCs before and after drug stimulation by using the current clamp mode of our amplifier with a stimulus waveform: I = 0 pA. The membrane passive properties (Rm, Gm and Cm) were consistently estimated by applying two step voltage pulses 75 ms long to −80 and −60 mV starting from a holding potential (HP) of −70 mV, in voltage clamp condition. Always in this mode, ionic currents were evoked by the following pulse protocols: outward K^+^ current activation was elicited by 1-s long voltage step pulses ranging from −80 to 50 mV applied in 10-mV increments from HP= −60 mV. Ca^2+^ current (I_Ca_) activation was evoked in the SMCs held at −80 mV, and 1-s long step pulses were applied in 10-mV increments from −70 to 50 mV; an interval of 20 s between episodes was given to allow recovery. I_Ca_ inactivation was investigated by a two-pulse protocol with a 1-s pre-pulse to different voltages followed by 1-s test pulse to 10 mV [28]. When we applied the two-pulse protocol, we used again a 20-s interval between stimulating episodes to consent to recovery. Capacitive, linear leak, and voltage-independent ionic currents were cancelled on-line using the P/4 procedure.

To evaluate the steady-state ionic current activation of voltage dependent channels, we used the following equation:

(1) Ia(V) = Gmax (V−Vrev)/{1 + exp[(Va−V)/ka]}

and the following was used for the steady-state current inactivation:

(2) Ih(V) = I/{1 + exp[−(Vh−V)/kh]},

where *Gmax* represents the maximal conductance for *Ia*; *Vrev* is the apparent reversal potential; *Va* and *Vh* are the voltages causing the half-maximal activation and inactivation, respectively; and *ka* and *kh* are steepness factors of activation and inactivation, respectively. 

### 4.5. Western Blotting

Fragments of gastric fundus from the control and the ADPN-treated mice were quickly minced and homogenized with a tissue homogenizer (Ing. Terzano, Milan, Italy) in cold lysis buffer of the following composition: 20 mM Tris/HCl (pH 7.4), 10 mM NaCl, 1.5 mM MgCl_2_, 5 mM EGTA, 2 mM Na_2_EDTA, added with 10× Sigmafast Protease Inhibitor Cocktail tablets and 100 mM sodium orthovanadate. Total protein content was measured spectrophotometrically using micro-BCA™ Protein Assay Kit (Pierce, IL, USA). Seventy μg of total proteins were electrophoresed by SDS–PAGE and blotted onto PVDF membranes (Millipore, Bedford, MA, USA). The membranes were incubated overnight at 4 °C with the following primary antibodies: rabbit polyclonal anti-AMPK (1: 1000; Cell Signaling, Danvers, MA, USA), rabbit polyclonal anti phosphoAMPK (Thr172), (pAMPK) (1:1000; Cell Signaling), and rabbit polyclonal anti-β-actin (1:20,000; Sigma Aldrich, Milan, Italy), assuming β-actin as control invariant protein. Specific bands were detected using rabbit peroxidase-labeled secondary antibodies (1:15.000 Vector, Burlingame, CA, USA) and enhanced chemiluminescent substrate. Densitometric analysis of the bands was performed using Scion Image Beta 4.0.2 image analysis software (Scion Corp., Frederick, MD, USA).

### 4.6. Immunofluorescence Analysis

Gastric tissue samples were rapidly excised and fixed by immersion in 4% paraformaldehyde, dehydrated in graded ethanol, embedded in paraffin, and cut in 5-μm thick sections. To evaluate AdipoR1 expression, double labeling immunofluorescence experiments were performed. The sections were deparaffinized, rehydrated, and boiled for 10 min in sodium citrate buffer (10 mM, pH 6.0, Bio-Optica, Milan, Italy) for antigen retrieval. To quench autofluorescence caused by the elastic fibers, the sections were incubated in 2 mg/mL glycine (AppliChem, Darmstadt, Germany) for 8 min at room temperature (RT). To minimize the unspecific binding, the sections were pre-incubated with 1.5% bovine serum albumin (Sigma Aldrich) for 20 min at RT and then incubated overnight at 4 °C with rabbit monoclonal anti-AdipoR1 antibody (1:400, Abcam, Milan, Italy) followed by goat anti-rabbit Alexa Fluor 488-conjugated IgG (1:350 Invitrogen, San Diego, CA, USA), for 2 h at RT. After the first incubation as described above, the sections were re-incubated overnight at 4 °C with goat polyclonal anti-αSMA (smooth muscle cell marker) antibody (1:200; Abcam), or with mouse monoclonal anti-UCH-L1 (neuronal marker) antibody (1:200; Santa Cruz Biotechnology, Texas, USA), or with chicken polyclonal anti-GFAP (glial cell marker) antibody (1: 500; Abcam). Then, the sections were incubated with the appropriate Alexa Fluor 568-conjugated IgG (1:350; Invitrogen) for 2 h at RT. To evaluate AMPK and pAMPK expression, gastric tissue sections from control and ADPN-treated mice were processed as above and incubated overnight at 4 °C with rabbit polyclonal anti-pAMPK antibody (1:50, Cell signaling) or rabbit polyclonal anti-AMPK (1:50; Cell Signaling) followed by goat anti-rabbit Alexa Fluor 488-conjugated IgG (1:175 Invitrogen) for 2 h at RT. After the first incubation, the sections were re-incubated overnight at 4 °C with chicken polyclonal anti-GFAP antibody (1: 500; Abcam), followed by donkey anti-chicken Alexa Fluor 568-conjugated IgG (1:350; Invitrogen) for 2 h at RT. Negative controls were performed by omitting the primary antibodies. The sections were mounted with Fluoroshied^TM^ mounting medium containing the nuclear marker DAPI (Sigma Aldrich). Images were obtained using an epi-fluorescence Olympus BX40 microscope coupled to analySIS∧B Imaging Software (Olympus, Milan, Italy) with 60× objectives.

### 4.7. Data Analysis and Statistical Tests

For functional experiments, amplitude of the responses is expressed as absolute values (grams) and measured when the maximal effect was reached. Basal tension was evaluated as changes in the recording baseline. 

For electrophysiological experiments, mathematical and statistical analysis of data was performed by pClamp6 (Axon Instruments) and Excel (Microsoft Office 2016, Microsoft corporation, Redmond, WA, USA). Data are collected from a representative, randomly selected portion of the total cell populations and the results of the experiments are expressed as mean ± SD. 

Student’s unpaired t-test was used to compare the average values of two datasets, assuming that values follow a normal distribution. One-way ANOVA was used for multiple comparisons followed by Bonferroni’s post hoc analysis. Statistical significance was set to *p* < 0.05. The number of muscle preparations/cells was designated by *n*.

### 4.8. Drugs

The following drugs were used: α-dendrotoxin (α-DTX, 10 nM); 4-aminopyridine (4-AP, 2 mM); barium chloride (BaCl_2_, 0.4 mM); chromanol (Chr, 50 µM); iberiotoxin (IbTx, 100 nM); mouse recombinant adiponectin (ADPN, 20 nM); nifedipine (10 µM); tetrodotoxin (TTX, 1 µM), carbachol (CCh, 1 μM), guanethidine (1 μM), L-NG-nitro arginine (L-NNA, 200 μM), dorsomorphin, (10 μM). All drugs were obtained from Sigma Chemical (St. Louis, MO, USA). 

Solutions were prepared on the day of the experiment, except for TTX and ADPN, for which stock solutions were kept stored at −20 °C.

Drug concentrations are referred as final bath concentrations. The chosen dose of ADPN is that previously reported to be effective in gastric preparations [5,6].

## Figures and Tables

**Table 1:** Passive membrane properties and normalized ion current amplitude (current response evoked by the +50 mV pulse) of gastric fundus SMCs in the different conditions.

	Cm (pF)	Gm (pS)	RMP (mV)	I_K_ (pA/pF)	I_Ca_ (pA/pF)
CTRL	13.5 ± 1.8	0.009 ± 0.002	−47.2 ± 5.0	17.1 ± 3.7	−20.3 ± 6.0
	(*n* = 52)	(*n* = 52)	(*n* = 50)	(*n* = 30)	(*n* = 20)
ADPN	24.7 ± 2.2 *	0.0175 ± 0.002	−59.4 ± 3.9 *	23.9 ± 2.1 *	−10.1 ± 2 *
	(*n* = 17)	(*n* = 16)	(*n* = 16)	(*n* = 9)	(*n* = 7)
L-NNA	12.3 ± 3.9 #	0.0072 ± 0.001	−47.0 ± 5.1 #	8.3 ± 2.9 *,#	−24.9 ± 4.1 #
	(*n* = 18)	(*n* = 18)	(*n* = 17)	(*n* = 10)	(*n* = 7)
L-NNA + ADPN	10.9 ± 3.1 #	0.0078 ± 0.001	−46.6 ± 4.3 #	7.8 ± 1.8 *,#	−10.0 ± 2.3 *,§
	(*n* = 18)	(*n* = 18)	(*n* = 17)	(*n* = 10)	(*n* = 7)
DORS	9.5 ± 6.7 #	0.005 ± 0.001	−39.2 ± 5.9 #	17.6 ± 3.0 #	−27.3 ± 4.1 #
	(*n* = 16)	(*n* = 13)	(*n* = 14)	(*n* = 7)	(*n* = 6)
DORS + ADPN	8.5 ± 2.4 #	0.007 ± 0.002	−35.7 ± 4.9 #	17.8 ± 2.8 #	−25 ± 2.3 #
	(*n* = 16)	(*n* = 13)	(*n* = 14)	(*n* = 7)	(*n* = 6)

One-way ANOVA with Bonferroni’s post hoc test was used for multiple comparisons. *, *p* < 0.05 significant difference from CTRL; #, *p* < 0.05 significant difference from ADPN; §, *p* < 0.05 significant difference from L-NNA. *n* represents the number of SMCs used (10 mice). Values are means ± SD.

**Table 2:** Boltzmann parameters of I_Ca,T_ and I_Ca,L_ activation and inactivation obtained in gastric SMCs in control condition (CTRL) and after adiponectin (ADPN) addition.

	I_Ca,T_		I_Ca,L_	
Parameters	CTRL	ADPN	CTRL	ADPN
I_Ca,p_/C_m_ (pA/pF)	1.7 ± 0.2	0.5 ± 0.1 ***	9.42 ± 0.6	5.2 ± 0.4 **
G_m_/C_m_ (pS/pF)	18 ± 5	5.5 ± 9 ***	46 ± 5.1	32 ± 3.8 **
V_thr_ (mV)	−54.8 ± 2	−53.0 ± 2	−50.2 ± 3	−48.7 ± 3
V_p_ (mV)	−25.1 ± 2	−30.2 ± 1.6 **	0.5 ± 0.07	10.2 ± 1 ***
V_a_ (mV)	−42.1 ± 3	−40.0 ± 2	−18.1 ± 2	−10.2 ± 2 ***
k_a_ (mV)	7.2 ± 0.4	7.1 ± 0.5	7.6 ± 0.3	8.1 ± 0.4
V_rev_ (mV)	76.5 ± 6	80.1 ± 7	79.4 ± 6	81.7 ± 7
V_h_ (mV)	−64.7 ± 6	−64.8 ± 6	−51 ± 4	−53 ± 5
k_h_ (mV)	4.5 ± 0.5	4.3 ± 0.4	7.5 ± 0.5	7.4 ± 0.5
t_p_ (ms)	5.5 ± 0.5	5.8 ± 0.5	22.7 ± 2	21.8 ± 3

ADPN decreases the normalized maximum peak size of the specific I_Ca,p_/C_m_ and the related G_m_/C_m_ for both T and L type Ca^2+^ current; moreover, it shifts the voltage values that elicits the maximal current in the I-V plots (Vp) and affects the I_Ca,L_ and I_Ca,T_ kinetics, influencing the Boltzmann parameters of activation (Va and ka) and inactivation (Vh and Kh). The subscript p indicates the peak value, V_thr_ is voltage threshold, V_rev_, is the apparent reversal potential, and t_p_ is peak time at the voltage eliciting the maximal current amplitude. All data are mean values ±SEM ** and *** indicate *p* < 0.01, and *p* < 0.001 ADPN vs. CTRL data, respectively (Student’s *t*-test). Data are from CTRL *n* = 20 cells, ADPN *n* = 7 cells, (5 mice).

## Abbreviations

ADPN	Adiponectin
C_m_	Cell linear capacitance
CTRL	Control
G_m_	Membrane conductance
G_m_/C_m_	Specific membrane conductance
HP	Holding potential
k_a_	Steepness factor of activation
k_h_	Steepness factor of inactivation
I_a_	Current activation
I_Ca_	Ca^2+^ current
I_K_	Voltage-dependent delayed rectifier K^+^ currents
RMP	Resting membrane potential
SMC	Smooth muscle cell
V_a_	Half-maximal activation voltage
V_h_	Half-maximal inactivation voltage
V_rev_	Apparent reversal potential
